# Concurrent de novo *MACF1* mutation and inherited 16p13.11 microduplication in a preterm newborn with hypotonia, joint hyperlaxity and multiple congenital malformations: a case report

**DOI:** 10.1186/s12887-024-04628-y

**Published:** 2024-08-16

**Authors:** Lanlan Mi, Ruen Yao, Weiwei Guo, Jian Wang, Guoqing Zhang, Xiuxia Ye

**Affiliations:** 1 Department of Neonatology, Shanghai Children’s Medical Center, Shanghai Jiao Tong University School of Medicine, Shanghai, China; 2 Department of Medical Genetics and Molecular Diagnostic Laboratory, Shanghai Children’s Medical Center, Shanghai Jiao Tong University School of Medicine, Shanghai, China

**Keywords:** *MACF1* mutation, 16p13.11 microduplication syndrome, Neonate, Congenital malformations

## Abstract

**Background:**

The *MACF1* gene, found on chromosome 1p34.3, is vital for controlling cytoskeleton dynamics, cell movement, growth, and differentiation. It consists of 101 exons, spanning over 270 kb. The 16p13.11 microduplication syndrome results from the duplication of 16p13.11 chromosome copies and is associated with various neurodevelopmental and physiological abnormalities. Both *MACF1* and 16p13.11 microduplication have significant impacts on neural development, potentially leading to nerve damage or neurological diseases. This study presents a unique case of a patient simultaneously experiencing a de novo *MACF1* mutation and a hereditary 16p13.11 microduplication, which has not been reported previously.

**Case presentation:**

In this report, we describe a Chinese preterm newborn girl exhibiting the typical characteristics of 16.13.11 microduplication syndrome. These features include developmental delay, respiratory issues, feeding problems, muscle weakness, excessive joint movement, and multiple congenital abnormalities. Through whole-exome sequencing, we identified a disease-causing mutation in the *MACF1* gene (c.15266T > C / p. Met5089Thr). Additionally, after microarray analysis, we confirmed the presence of a 16p13.11 microduplication (chr16:14,916,289 − 16,315,688), which was inherited from the mother.

**Conclusions:**

The patient’s clinical presentation, marked by muscle weakness and multiple birth defects, may be attributed to both the de novo *MACF1* mutation and the 16p13.11 duplication, which could have further amplified her severe symptoms. Genetic testing for individuals with complex clinical manifestations can offer valuable insights for diagnosis and serve as a reference for genetic counseling for both patients and their families.

**Supplementary Information:**

The online version contains supplementary material available at 10.1186/s12887-024-04628-y.

## Background

*MACF1* (Microtubule actin crosslinking factor 1) is a large protein expressed in various tissues, with high levels found in the heart, skeletal muscles, intestine, colon, and moderate levels in the brain and lungs [1]. Abnormal expression of *MACF1* can result in dysfunctional tissue development and organ function across multiple organs. *MACF1* mutations are associated with neuromuscular disorders characterized by progressive spastic tetraplegia, dystonia, joint contractures, feeding difficulties, and developmental delays [2]. Additionally, *MACF1* plays a role in regulating osteoblast proliferation and differentiation [3], and its aberrant expression is linked to the development of several tumors due to increased cell proliferation [4].

The 16p13.11 microduplication is a recurring genetic anomaly found not only in individuals with neurological impairments but also in healthy individuals [5]. Clinical manifestations of this duplication include multiple congenital abnormalities and primarily neurodevelopmental disorders such as speech delays, learning disabilities, attention-deficit/hyperactivity disorder, intellectual disabilities, developmental delays, and autistic spectrum disorder (ASD) [6–9]. Furthermore, there is a notable increased risk of cardiovascular diseases associated with this duplication [6, 10].

In this study, we present the case of a female patient who possesses a de novo *MACF1* mutation alongside a 16p13.11 duplication spanning 1300 kb. This patient exhibits global developmental delay, respiratory distress, feeding difficulties, and multiple dysmorphic features. We also explore the combined effects of both genetic abnormalities on the patient’s phenotype.

## Case presentation

The patient is a female born to healthy, non-consanguineous parents with an unremarkable family history. She was conceived through in vitro fertilization-embryo transplantation (IVF-ET) and was delivered via caesarean section at 33^+5^ weeks of gestation due to fetal growth restriction, preeclampsia, and a prior hysteromyoma surgery. At birth, she weighed 1595 g (10th percentile) and had an occipitofrontal circumference of 28 cm (5th percentile), indicating she was smaller than expected for her gestational age.

The patient exhibited distinctive facial features, including a broad forehead, arched eyebrows, sunken eye sockets, small and slanted eye openings, corneal edema, a flat nasal bridge, and upturned nostrils. Additionally, she had a flat chest, an increased palmar crease on her right hand, and anteriorization of the anus close to the labia majora, covered by a white film. Neurologically, she displayed hypotonia, reduced light reflex in both eyes, absence of crying, decreased motor activity, and intracranial hemorrhage (Level IV).

Following birth, the patient experienced severe breathing difficulties, necessitating immediate intubation. She was subsequently transferred to the NICU at Shanghai Children’s Medical Center, where she required invasive mechanical ventilation for the first 18 days of life. Her ECG indicated a slightly prolonged QT interval, and cardiac ultrasound revealed a patent ductus arteriosus (0.15 cm), an atrial septal defect (0.25 cm), and stable hemodynamics.

**Fig. 1:**
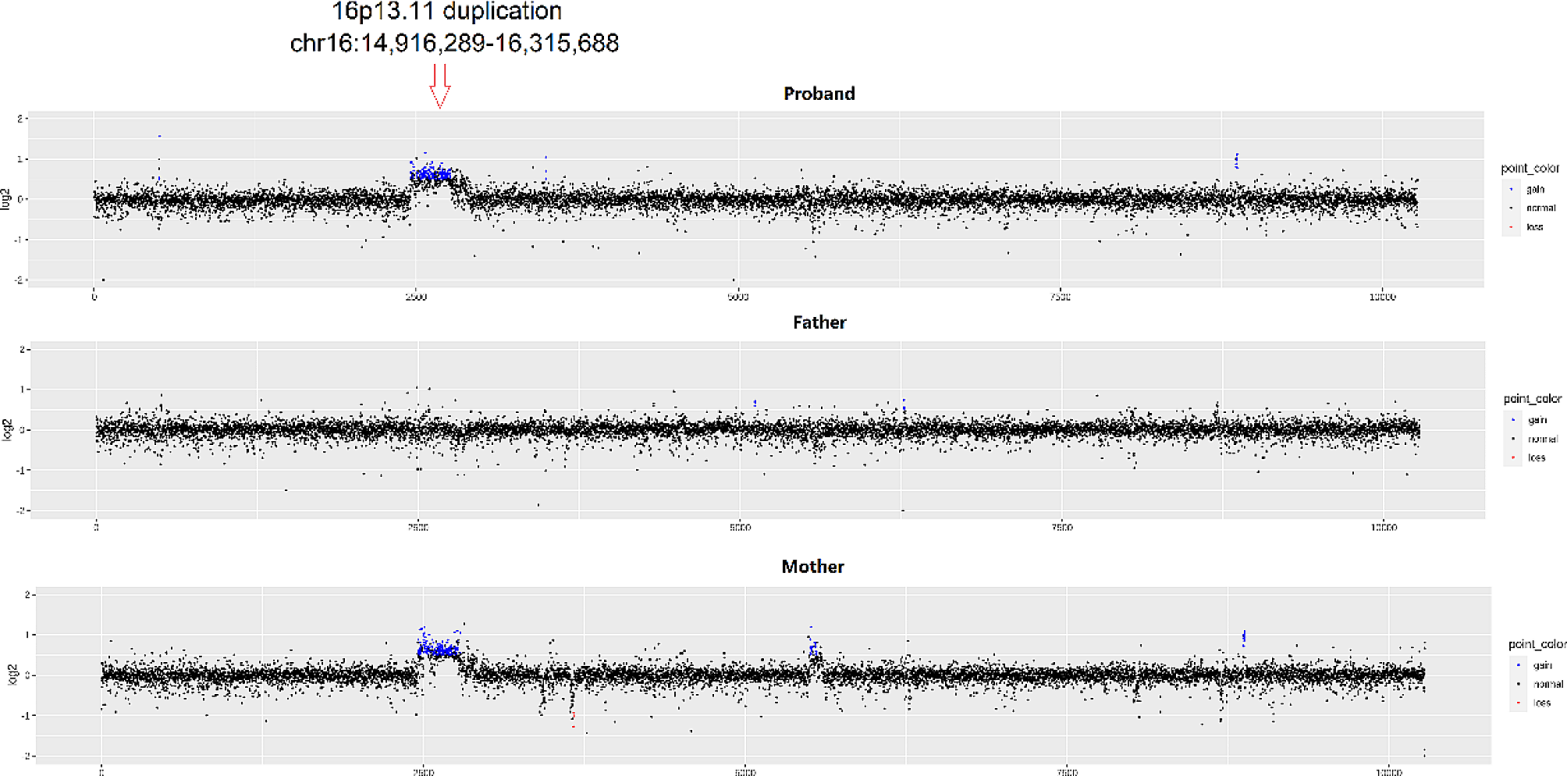
In the first chromosome, representing the proband (the patient), and the following two chromosomes, representing her father and mother, the red arrow points to the segment of chromosome 16 (chr16:14,916,289 − 16,315,688) microduplication

**Fig. 2:**
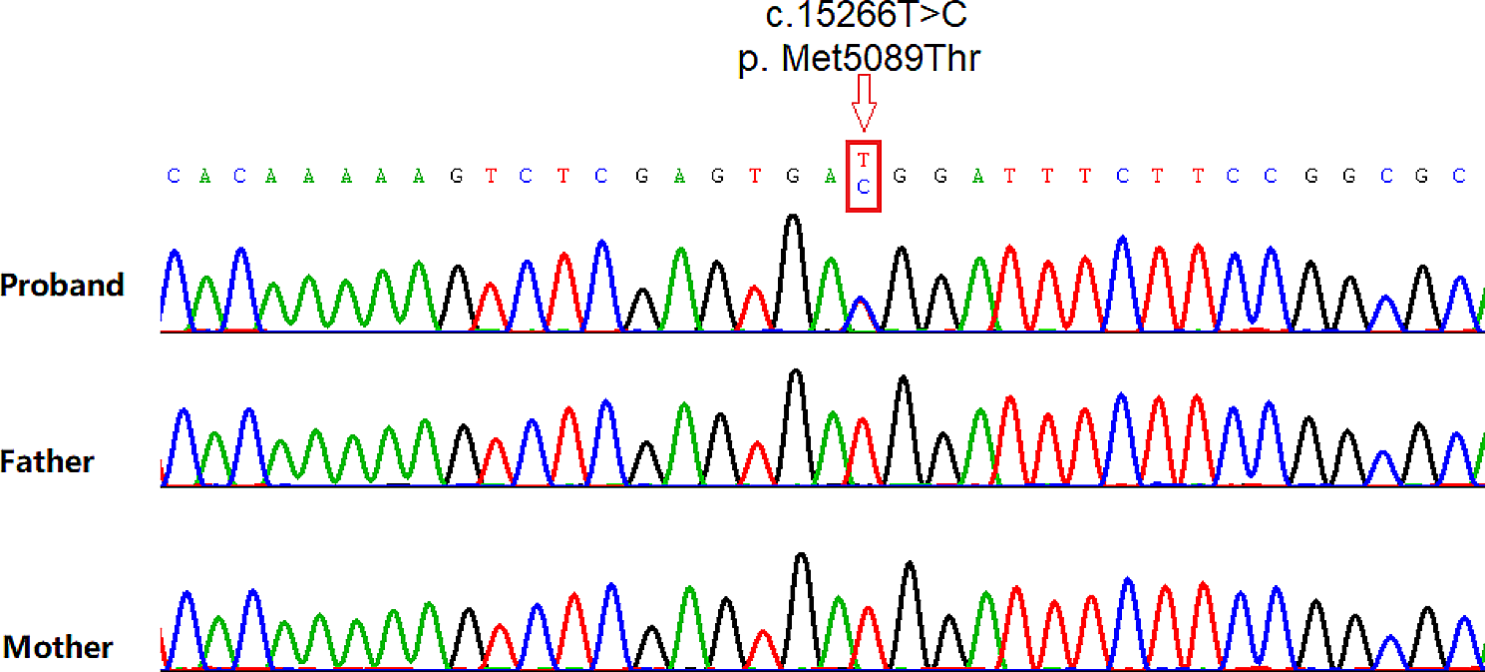
The initial gene chain corresponds to the proband (the patient), and the subsequent two gene chains correspond to her father and mother, respectively. The red arrow indicates the presence of the *MACF1* (c.15266T > C / p. Met5089Thr) mutation

The patient also developed significant feeding intolerance and gastric retention. Further assessments indicated delayed gastric emptying and the possibility of digestive tract malformation. Notably, she displayed excessive joint mobility, with her leg angle exceeding 180 degrees.

a-CGH identified a duplication on the short arm of chromosome 16 (chr16:14,916,289 − 16,315,688), which was inherited from the mother (Fig. 1). Sanger sequencing confirmed the wild-type status of the *MACF1* gene (c.15266T > C / p. Met5089Thr) mutation in the parents, with the proband being heterozygous (Fig. 2). Machine learning-based pathogenicity prediction of the *MACF1* variants indicated their deleterious nature (0.98, 0.99) according to Alpha Missense and DANN [11, 12]. Other variants in the *MACF1* gene, apart from the de novo mutation, were excluded due to insufficient evidence of pathogenicity (Supplementary Table 1).

## Discussion and conclusions

*MACF1*, a member of the cytoskeleton cross-linking protein family, plays a crucial role in regulating the dynamics of the cytoskeleton network. It exhibits high expression levels in neuronal tissues and the foregut of embryonic day 8.5 (E8.5) embryos, as well as in the head fold and primitive streak of E7.5 embryos [13]. The presence of the microtubule-binding growth-arrest-specific 2 domain variant of *MACF1* is a unique and likely contributor to brain abnormalities, particularly severe dorsal-ventral stenosis of the brainstem, primarily occurring in the pontine and midsagittal regions of the cerebellar eminence [14]. *MACF1*’s role in controlling neurite growth and branching involves the regulation of actin and microtubule alignment and stability, mediating GSK3 signals. In mice, the deletion of *MACF1* has been associated with abnormalities in dendritic differentiation in cortical and hippocampal neurons [15]. In humans, *MACF1* mutations may be inherited in an autosomal recessive manner, leading to spectraplakinopathy characterized by progressive spastic tetraplegia, dystonia, joint contractures, feeding difficulties, and developmental delays [2]. *MACF1* is also considered a potential novel recessive epilepsy gene [16]. It is associated with congenital myasthenia [17] and congenital pulmonary airway malformations [18]. These factors collectively account for our patient’s intrauterine growth retardation, hypotonia, flat chest, abnormal breathing, and feeding difficulties.

Cranial nerve defects resulting in dysphagia were observed in four children, while one child exhibited asymmetric facial movements, three had strabismus, two had cortical visual impairment, and one had left optic-nerve hypoplasia [14]. Similar findings were reported in another Japanese child [19]. May Simera et al. [20] demonstrated that the deletion of *MACF1* in mice led to impaired retinal delamination and lamination, as well as halted photoreceptor maturation. Consistent with these animal models, our patient experienced difficulty feeding and displayed reduced light reflex in both eyes. Furthermore, *MACF1*’s impact on osteoblast proliferation, differentiation, and bone formation may explain the bone deformities and excessive joint mobility observed in patients [21]. Due to its expression in multiple organs and tissues, abnormal *MACF1* expression is implicated in the development of various tumors [22, 23].

In addition to the de novo *MACF1* mutation, our patient also carried the maternal 16p13.11 duplication. Copy number variations (CNVs) like this are typically inherited from seemingly healthy parents, often carriers of balanced translocations [24]. Homozygous forms of these rearrangements have been observed in individuals with various behavioral phenotypes, including intellectual disability, developmental delay, speech delay, and emotional and behavioral disorders such as attention-deficit/hyperactivity disorder and ASD [25, 26]. Additionally, neurodevelopmental abnormalities encompass gross motor delay, epilepsy, and schizophrenia [26]. Some patients with 16p13.11 microduplication have presented with abnormal brain MRI findings, as well as cardiac and aortic/pulmonary malformations [6]. Notably, certain individuals have exhibited prominent skeletal features like craniosynostosis, polydactyly, and excessive joint mobility [27].

Other medical conditions associated with 16p13.11 microduplication include a range of congenital abnormalities of varying severity, including distinctive facial features [28] such as deep-set eyes [29], upslanting palpebral fissures, preauricular skin tags [26], and low-set ears [27]. Microcephaly or macrocephaly, craniosynostosis, polydactyly, tall stature, pes planus, and vision issues like strabismus, myopia, and amblyopia have also been reported [24]. Some patients have shown intestinal malrotation and absent 12th ribs [30, 31].

The neonatal neurodevelopmental phenotype associated with this microduplication often includes hypotonia, feeding difficulties, and respiratory problems [6, 24]. Unfortunately, our newborn patient only survived for 18 days after birth and displayed multiple congenital malformations, hypotonia, excessive joint mobility, feeding difficulties, respiratory problems, and cardiac malformations, consistent with previous reports. However, her phenotype was more severe due to the combined effects of the *MACF1* mutation and the 16p13.11 microduplication. In continuous genetic syndromes, while naturally conceived children may have genetic abnormalities, assisted reproductive techniques (ART) can increase the likelihood of epigenomic defects [32, 33]. It’s important to consider the potential impact of ART on our patient’s phenotype. Epigenetics is influenced by various factors, and even overlapping or very similar genetic changes can lead to phenotypic variations, resulting in varying disease severity [34]. Therefore, studying more cases can contribute to a better understanding of the genotype-phenotype correlation.

The varied clinical manifestations seen in 16p13.11 microduplication syndrome may be attributed to different genes within this region, which contains 13 distinct OMIM genes [28]. These genes include *ABCC1, ABCC6, CEP20, MARF1, MPV17L, MYH11, NDE1, NOMO1, NOMO3, NPIPA1, NTAN1, PDXDC1*, and *RRN3.* Notably, NDE1 plays a crucial role in microtubule organization, mitosis, and neuronal migration [35]. Changes in its dosage can result in secondary genomic damage to cortical neurons [36], making it a prime candidate gene for the neurological and behavioral phenotypes observed in affected patients [37, 38]. Additionally, miR-484 also plays a vital role in the neurocognitive phenotype [25], and MYH11 has been identified in familial cases of aortic aneurysms with an elevated risk of dissection [10, 39]. However, the roles and mechanisms of the other genes in the 16p13.11 duplication syndrome remain unclear, necessitating further research.

Many of the clinical features observed in our patient align with those found in previous cases affected by either *MACF1* mutation or 16p13.11 microduplication syndrome. Moreover, our patient’s phenotype was more severe, likely due to the combined effects of both genetic abnormalities. Interestingly, the presence of an increased palmar crease is the first reported case in 16p13.11 microduplication syndrome. Expanding our understanding of the phenotypes associated with 16p13.11 duplication syndrome and their potential relationships with specific genetic and genomic loci will contribute to further geno-phenotype research. Patients with concurrent genomic variants on various scales are at risk of being missed or misdiagnosed. An integrated genetic testing and analysis approach, supported by a detailed description of the phenotype, can enhance diagnostic yield and lead to novel discoveries in clinical diagnostics [40, 41]. This report underscores the importance of thoroughly analyzing the phenotypes of patients with complex genotypes and provides additional insights and characterizations. We hope that this report can aid in genetic counseling and further research in this field.

### Electronic supplementary material

Below is the link to the electronic supplementary material.

Supplementary Material 1

## Data Availability

The raw sequence data reported in this paper have been deposited in the Genome Sequence Archive (Genomics, Proteomics & Bioinformatics 2021) in National Genomics Data Center (Nucleic Acids Res 2022), China National Center for Bioinformation / Beijing Institute of Genomics, Chinese Academy of Sciences (GSA-Human: HRA006654) that are publicly accessible at https://ngdc.cncb.ac.cn/gsa-human.

## Abbreviations

*MACF1*: Microtubule action crossing factor 1
ASD: Autistic spectrum disorder
CNV: Copy number variation
