# Supplementary material for: Concurrent de novo MACF1 mutation and inherited 16p13.11 microduplication in a preterm newborn with hypotonia, joint hyperlaxity and multiple congenital malformations: a case report
Source: BMC Pediatr. 2024 Aug 16;24:528. doi: 10.1186/s12887-024-04628-y (PMC11328432; doi:10.1186/s12887-024-04628-y)
Supplement: Supplementary file 1 — Supplementary Material 1 [file 12887_2024_4628_MOESM1_ESM.docx]

Supplementary table 1

Other rare variants detected in the pedigree

| Gene name | position | cDNA | protein | Genotype | ENST_Number | Depth | Revel_Score | gnomad_exon_AF | gnomad_genome_AF |
| --- | --- | --- | --- | --- | --- | --- | --- | --- | --- |
| *CDON* | chr11-125851003-g-t | c.3217C>A | p.His1073Asn | het;het;- | exon17 | 86/52(0.38);111/93(0.46);- | 0.271 | - | - |
| *CITED2* | chr6-139694436-t-a | c.646A>T | p.Asn216Tyr | het;het;- | exon2 | 92/105(0.53);124/126(0.5);- | 0.44 | - | - |
| *COL11A1* | chr1-103343637-c-g | c.5359G>C | p.Asp1787His | het;-;het | exon67 | 24/27(0.53);-;36/29(0.45) | 0.937 | - | - |
| *DIAPH3* | chr13-60557954-g-c | c.1429C>G | p.Pro477Ala | het;-;het | exon13 | 14/9(0.39);-;8/8(0.5) | 0.75 | - | - |
| *EHHADH* | chr3-184910909-t-c | c.1277A>G | p.His426Arg | het;het;- | exon7 | 83/73(0.47);98/93(0.49);- | 0.213 | - | - |
| *JAG1* | chr20-10621777-t-g | c.3032A>C | p.Glu1011Ala | het;-;het | exon24 | 70/86(0.55);-;128/115(0.47) | 0.402 | - | - |
| *SACS* | chr13-23911789-a-c | c.6226T>G | p.Leu2076Val | het;-;het | exon10 | 23/10(0.3);-;31/31(0.5) | - | - | 0.00000801 |
| *WNK1* | chr12-992601-t-g | c.4310T>G | p.Phe1437Cys | het;-;het | exon16 | 19/12(0.39);-;51/29(0.36) | 0.786 | - | - |
